# Predictive Model of Early Spontaneous Ductus Arteriosus Closure Based on Neonatologist Performed Echocardiography in Preterm Infants

**DOI:** 10.3389/fped.2021.644519

**Published:** 2021-02-26

**Authors:** María Carmen Bravo, Rebeca Sánchez, Ana Isabel Blanco, Itsaso Losantos, Adelina Pellicer

**Affiliations:** ^1^Department of Neonatology, La Paz University Hospital, Madrid, Spain; ^2^Division of Statistics, La Paz University Hospital, Madrid, Spain

**Keywords:** ductus arteriosus, echocardiography, preterm infant, ibuprofen, NPE

## Abstract

**Background:** Patent ductus arteriosus (PDA) treatment remains controversial. Modeling on the predictive capacity of early spontaneous PDA closure would help in decision-making.

**Aim:** To design a predictive model of early spontaneous PDA closure.

**Methods:** As part of a trial to assess efficacy and safety of two ibuprofen treatment schemes for PDA, infants below 29 weeks' gestation were scanned between 18 and 72 h of birth, and serially if indicated. PDA treatment was decided based on echocardiography signs of lung overflow or systemic hypoperfusion and clinical criteria. A PDA score that included the echocardiographic parameters significantly associated with treatment prescription was retrospectively applied. Perinatal variables and screening score were included in a backwards elimination model to predict early spontaneous closure.

**Results:** Among 87 eligible infants (27 weeks' gestation; age at screening 45 h), 21 received ibuprofen at 69 h of life [screening score = 7 (IQR = 5–8.5); score at treatment = 9 (IQR = 8–9)], while 42 infants had conservative management, [screening score = 1 (IQR = 0–4)]. Twenty four infants were excluded (ibuprofen contraindication, declined consent or incomplete echocardiography). Screening score showed an AUC = 0.93 to predict early spontaneous PDA closure, [cut-off value = 4.5 (sensitivity = 0.90, specificity = 0.86)]. The predictive model for early spontaneous PDA closure followed the equation: Log (p/1-p) = −28.41 + 1.23^*^ gestational age −0.87^*^ PDA screening score.

**Conclusions:** A predictive model of early spontaneous PDA closure that includes gestational age and the screening PDA score is proposed to help clinicians in the decision- making for PDA treatment. In addition, this model could be used in future intervention trials aimed to prevent PDA related morbidities to improve the eligibility criteria.

## Introduction

The patent ductus arteriosus (PDA) is associated with several morbidities including intraventricular hemorrhage (IVH), necrotizing enterocolitis (NEC), bronchopulmonary dysplasia (BPD), and death as a result of pulmonary over-circulation and systemic hypoperfusion (1–6). Different management strategies have been studied in the last years, such as prophylactic treatment, early targeted treatment, treatment of a clinically symptomatic PDA, and the conservative approach of “watchful waiting” (7–12). The reported spontaneous closure rate reaches 73% at 2 months of postnatal age in extremely low gestational age neonates who survive beyond the neonatal period (11). Yet, none of these approaches have shown a reduction in the prevalence of PDA-related morbidities. This lack of benefit may be due to the fact that some infants with borderline ductal shunts (non-significant) were included in the treatment group in these trials (9). In addition, in case of active treatment is decided, there is not consensus on “how early” should treatment be started, as severe co-morbidities like IVH or pulmonary hemorrhage occur very early after birth and would be only prevented by prophylactic or very early targeted treatment (before 12 h form birth) (9, 13). Moreover, pharmacological efficacy of the cyclooxygenase inhibitors is inversely related to postnatal age at treatment administration (14).

Serial neonatologist performed echocardiography (NPE) facilitate identification of a hemodynamically significant PDA (hsPDA), and may also inform on the likelihood of spontaneous PDA closure. We hypothesized that an early predictive NPE model for spontaneous PDA closure beyond the first day of life will avoid overtreatment of babies with closing PDA patterns.

This retrospective study, that combines systematic serial NPE and prospective clinical data recordings, aims to build up a model to classify two populations of preterm infants early in the clinical course: those with a closing PDA pattern that will not be subsidiary of an active PDA treatment, from those with hsPDA and pathophysiologically related (lung hyperperfusion or systemic hypoperfusion) co-morbidities, therefore beneficiaries of an active early PDA treatment.

## Method

Infants with <29 completed weeks of gestation underwent echocardiographic assessment between 18 and 72 h of birth, as part of the routine care at NICU, in the foregoing discussion named as screening NPE. Those with NPE criteria of hsPDA were considered eligible for a multicentre randomized controlled trial to compare the efficacy and safety of two different intravenous ibuprofen treatment schemes (15-min bolus infusion vs. 24-h continuous infusion). Pharmacological PDA closure at this stage was always indicated by the attending physician based on echocardiography data and the infant's clinical condition. Serial scans were performed until PDA closure was confirmed or hospital discharge, whatever came first.

A hsPDA was defined by NPE if PDA showed a diameter larger than 1.5 mm and echocardiographic signs indicating pulmonary overflow, systemic hypoperfusion, or both were present, following the latest recommendations of the European Special Interest Group “Neonatologist Performed Echocardiography” (15). Infants were excluded in the case ibuprofen treatment was contraindicated or had no informed consent signed. If an open duct was not judged as hsPDA, or even in the case of pulmonary overflow or systemic hypoperfusion the attending physician decided a “watchful waiting,” serial scans were undertaken to confirm PDA closure or need for treatment prescription.

Infants who received ibuprofen for PDA closure at any time during the neonatal period formed the ibuprofen treatment (IB-T) group, and those who did not received any pharmacological treatment to close PDA formed the conservative management (CM) group.

The study protocol was approved by the Ethics Committee for Human Studies at La Paz University Hospital and the Spanish Medicines Agency at the National Ministry of Health (Eudract Code: 2016-002974-11 and ClinicalTrials.gov code: NCT04282941).

### NPE Studies

An Aplio 500 ultrasound scanner (Toshiba Medical Systems B.V, Netherlands) with a multifrequency phased array transducer 7–10 Mhz was used. Structural normality of the heart was established on the initial scan and standardized criteria for PDA hemodynamic significance were followed (15–18).

Transductal diameter was measured from a high left-sided parasternal “ductal” view to obtain a clear 2D image of the ductus arteriosus. The probe was placed in a true sagittal plane to the left of the sternum with the marker pointing toward the head to obtain the ductal view. The ductal diameter was measured at the site of maximum constriction (shortest diameter) at the pulmonary end, using color Doppler imaging, increasing the gain until background noise was suppressed, and expressed in mm. Ductal maximum and minimum velocity (Vmax/Vmin) ratio, was measured applying pulsed-wave Doppler (PWD) at the pulmonary end of the ductal view. Antegrade left pulmonary artery (LPA) diastolic flow, was visualized from a high parasternal short axis view using color Doppler. End-diastolic velocity was measured by PWD in the LPA tracing the Doppler signal and expressed in cm/s. Left atrial/aortic ratio was measured from the parasternal long axis view using M-mode at end-diastole, positioning the probe perpendicular to the aorta at the level of the aortic valve. To calculate the left ventricular output (LVO), aortic root diameter was measured at the hinges of the aortic valve leaflets, from the long axis parasternal view. The velocity time integral (VTI) of the ascending aorta was obtained by PWD from the apical 5-chamber view, aligning the probe to become parallel to flow direction, without angle of correction. Three consecutive Doppler wave forms were averaged and used to estimate the VTI. LVO (mL/kg/min) was derived from the equation: [(aortic diameter^2^/4) * π * *VTI* * heart rate]/ weight. Superior vena cava (SVC) diameter was calculated from the parasternal long axis view with the ultrasound beam in a true sagittal plane and angled to the right of the ascending aorta. Maximum and minimum diameters of three to five consecutive cardiac cycles, obtained by M-mode at the point the SVC starts to open into the right atrium, were averaged. The VTI of the SVC was measured from a subcostal window and the angle of insonation was minimized by maneuvering the transducer inferiorly to allow visualization of the maximal amount of flow within the SVC before entry into the right atrium. The pulsed Doppler recording was made at the junction of the SVC and the right atrium. The averaged VTI from 5 to 10 consecutive cardiac cycles was obtained. SVC flow (mL/kg/min) was derived from the equation: [(mean SVC diameter^2^/4) * π * VTI * heart rate] / weight. Then, the LVO/SVC flow was calculated.

E wave/A wave ratio was obtained from a four-chamber view with the probe positioned on the apex of the heart with the marker facing the left side. The probe was tilted to the right shoulder and the pulsed Doppler range gate was set slightly below the mitral valve annulus.

NPE were always performed by the same study team (MCB, RS, and AIB) for whom inter and intra-observer variability study was conducted ahead to the trial start on a sample of 57 scans. The intraclass correlation coefficient ranged between 0.88 and 0.98 (*p* < 0.001) for all the variables that were explored.

### Statistical Analysis

The data were analyzed using the SAS version 9.4 (SAS Institute Inc. 2013). The quantitative perinatal data and echocardiographic variables were expressed as median and interquartile range (IQR), and the qualitative data as count and percentage (%). IB-T group and CM group were compared using the Mann-Whitney U or Kruskal-Wallis-test and Fisher's exact or χ^2^-test for quantitative and qualitative variables, respectively.

A multiple regression analysis was performed to evaluate the association between perinatal and NPE variables with ibuprofen treatment. Different combinations of the NPE variables were used to calculate the best PDA score to categorize PDA disease and to predict early spontaneous PDA closure, adapted from that proposed by Sehgal et al. (19) for ductal disease severity staging. A receiver operating characteristics curve was constructed to assess the ability of PDA score at screening NPE to predict early spontaneous PDA closure.

The best model for PDA score was retrospectively calculated at screening NPE and at the time of treatment administration. In addition, a backwards elimination method was applied to create the best predictive model of early spontaneous PDA closure, based on the probability of the Wald statistic. A full (saturated) model including all the perinatal variables statistically associated with spontaneous closure (positive and negative associations) from the multiple regression model was created. Then, at each step the analysis gradually eliminates variables from the regression model to find a reduced model that best explains the data.

All the statistical analyses were considered bilateral, and values of *p* < 0.05 were considered significant.

## Results

**Figure 1:**
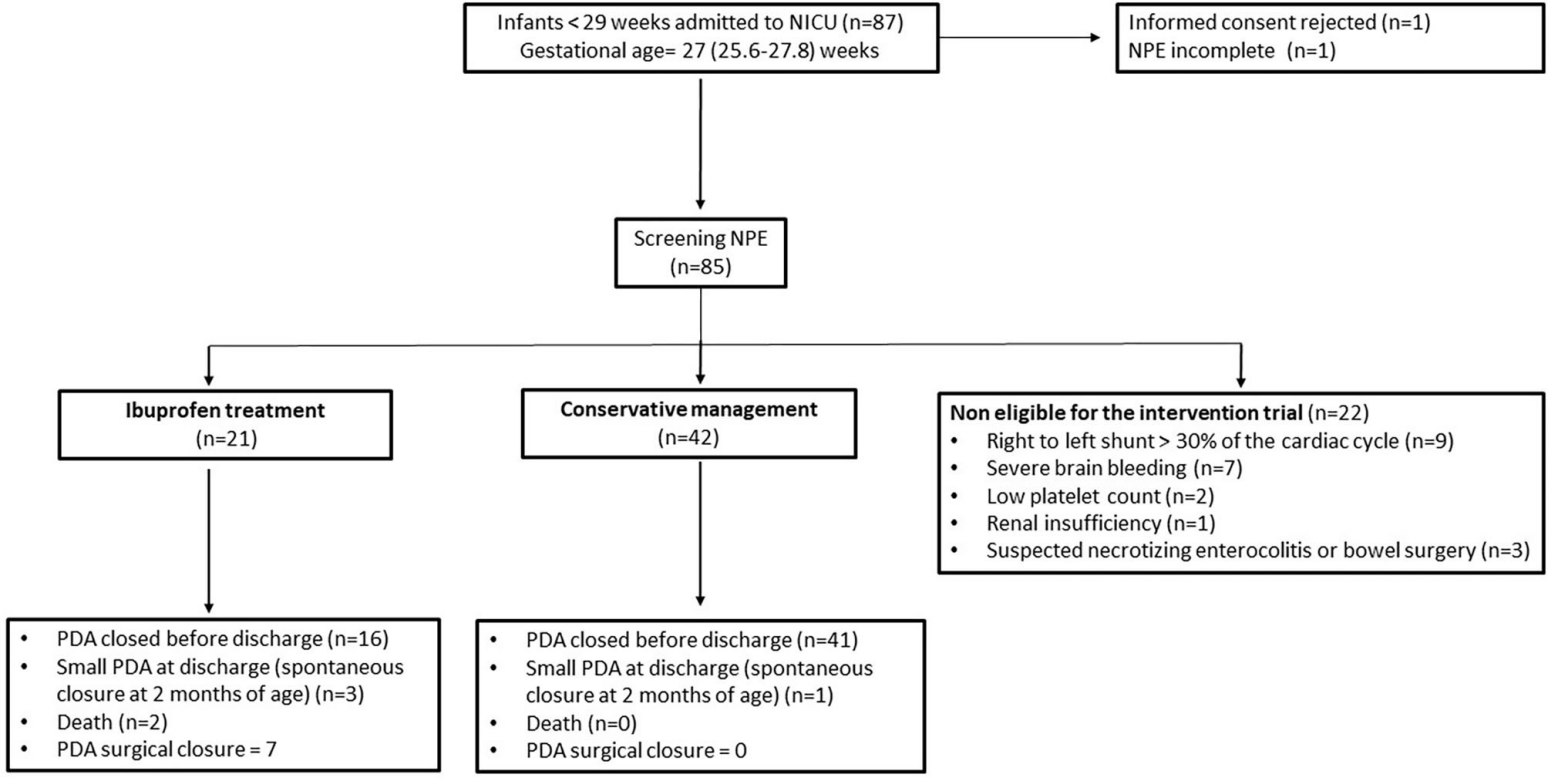
Patient flowchart.

From July 2018 to April 2020, 87 infants of 27 (25.6–27.8) weeks of gestation were eligible for the study (Figure 1). Of them, 85 underwent screening NPE assessment at a median postnatal age of 45 (24–49) h. Informed consent was declined in one infant, and an additional infant was not included due to incomplete data on screening NPE. Twenty one infants who were identified as having hsPDA by NPE received ibuprofen treatment (IB-T group) at a median postnatal age of 69 (52–158) h, while 42 infants who did not show echocardiographic signs of hsPDA were not treated and formed the CM group. Among the former, treatment was indicated in 7 infants as a result of the screening NPE, while in the remaining 14, a second NPE was needed for decision-making.

Twenty two additional infants underwent screening NPE but were not considered eligible for the intervention trial due to persistent pulmonary hypertension (*n* = 9) or other clinical conditions (*n* = 13). This precluded to have a complete systematic approach by NPE in this subset of infants and accordingly are not included in this retrospective analysis. Details of the study population chart flow are depicted on Figure 1.

Infants in the IB-T group had lower gestational age (*p* < 0.001), birth weight (*p* = 0.003), and 5-min Apgar (*p* = 0.04), showed higher rates of advanced resuscitation at birth (*p* = 0.03), and had larger transductal diameter (*p* < 0.001), higher ductal Vmax/Vmin ratio (*p* < 0.001) and higher LPA diastolic velocity (*p* < 0.001). In addition, reverse descending aorta diastolic flow was more prevalent at screening NPE in infants in the IB-T group than in those of the CM group (*p* < 0.001).

**Figure 2:**
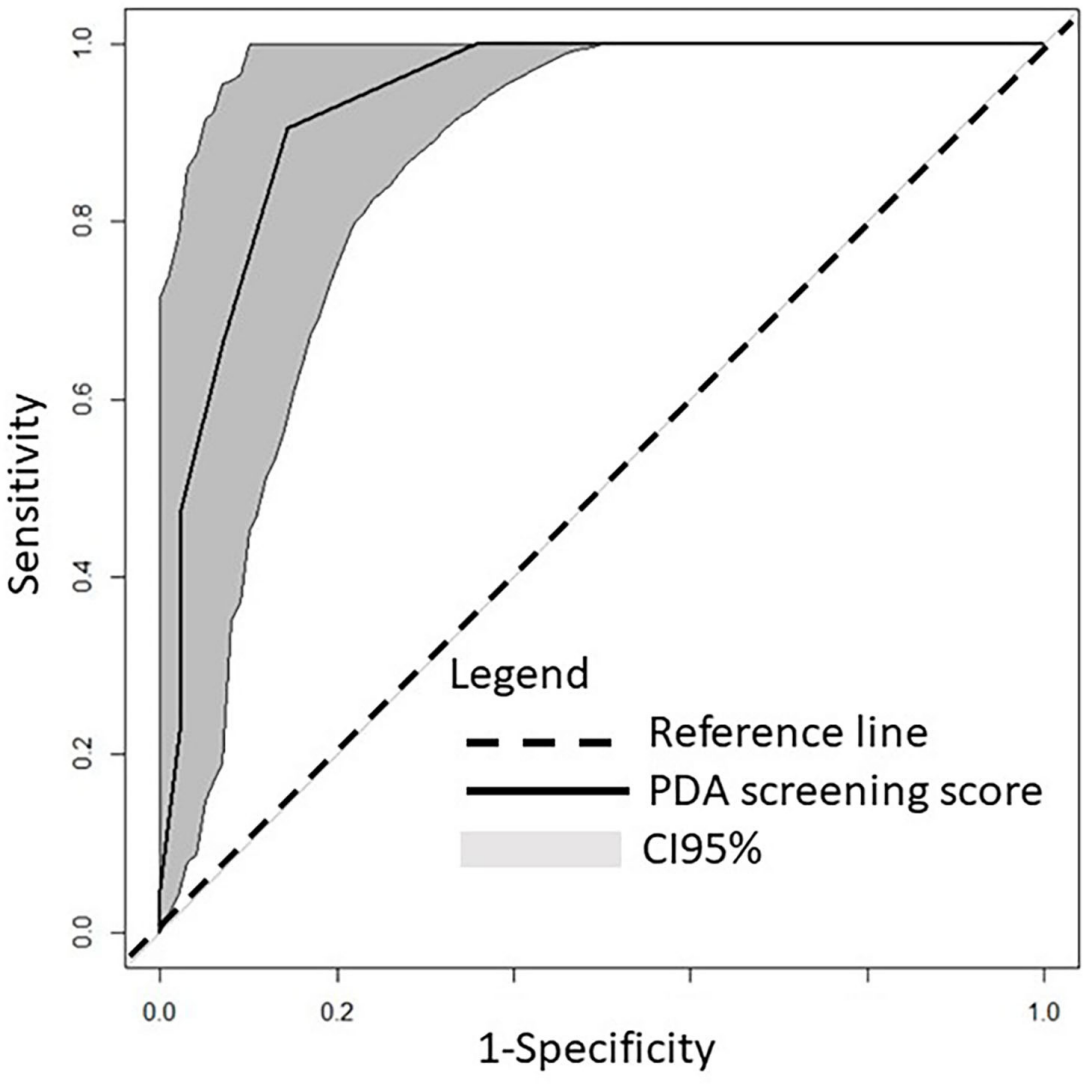
Receiver operating characteristics curve of the ability of screening PDA score to differentiate between PDA ibuprofen treatment group and early PDA spontaneous closure (conservative management group).

The predictive capacity of screening NPE for early spontaneous PDA closure was analyzed by different models of PDA score. This analysis yielded that the best model of PDA score included only the echocardiographic variables statistically associated with the CM group (Tables 1, 2). It showed an AUC = 0.93 (95% CI 0.88–0.99), with PDA score cut-off value <4.5 with a sensitivity of 0.90 (95% CI 0.71–0.97), specificity of 0.86 (95% CI 0.72–0.93), positive predictive value of 0.76 (95% CI 0.59–0.87), and negative predictive value of 0.95 (95% CI 0.83–0.98) (Figure 2).

**Table 1:** Proposed predicted models of early spontaneous PDA closure.

**Predicted model**	**AUC (95% CI)**	**Cut-off value**	**Sensitivity (95% CI)**	**Specificity (95% CI)**	**PPV (95% CI)**	**NPV (95% CI)**
Model 1	0.89 (0.82–0.97)	6.5	0.86 (0.65–0.95)	0.78 (0.64–0.88)	0.67 (0.52–0.78)	0.92 (0.79–0.97)
Model 2	0.89 (0.82–0.97)	6.5	0.80 (0.60–0.92)	0.80 (0.66–0.90)	0.68 (0.52–0.80)	0.89 (0.77–0.95)
Model 3	0.90 (0.83–0.97)	4.5	0.90 (0.71–0.97)	0.74 (0.59–0.85)	0.63 (0.50–0.74)	0.94 (0.80–0.98)
Model 4	0.89 (0.81–0.97)	6.5	0.86 (0.65–0.95)	0.81 (0.67–0.90)	0.69 (0.54–0.81)	0.92 (0.79–0.97)
Model 5	0.93 (0.88–0.99)	4.5	0.90 (0.71–0.97)	0.86 (0.72–0.93)	0.76 (0.59–0.87)	0.95 (0.83–0.98)

*Model 1 includes all the echocardiographic variables (transductal diameter, ductal velocity Vmax/Vmin ratio, antegrade LPA diastolic flow, Left atrial: aortic ratio, LVO/SVC flow ratio, E wave/A wave ratio and reverse descending aorta diastolic velocity). Model 2 includes all the echocardiographic variables but E/A ratio. Model 3 includes all the echocardiographic variables but left atrial/aortic ratio. Model 4 includes all the echocardiographic variables but LVO/SVC flow. Model 5 includes only the echocardiographic variables significantly associated with conservative management (transductal diameter, ductal velocity Vmax/Vmin ratio, antegrade LPA diastolic flow and reverse descending aorta diastolic velocity)*.

*Transductal diameter (mm): <1.5 = score 1; 1.5–3 = score 2; >3 = score 3; Ductal velocity Vmax/Vmin ratio (m/s): <1.5 = score 1; 1.5–2 = score 2; >2 = score 3. Antegrade LPA diastolic flow (cm/s): <30 = score 1; 30–50 = score 2; >50 = score 3; Left atrial: aortic ratio: <1.4:1 = score 0; 1.4–1.6:1 = score 2; >1.6:1 = score 3; LVO/SVC flow ratio: <4 = score 0; ≥4 = score 3; E wave/A wave ratio: <1 = score 0; 1–1.5 = score 2; >1.5 = score 3*.

*AUC, area under the curve; CI, confidence interval; PPV, positive predictive value; NPV, negative predictive value*.

**Table 2:** Ductal disease stage^*^ (Model 5).

	**Modality/position of sample gate**	**Score 0**	**Score 1**	**Score 2**	**Score 3**
Transductal diameter, mm	Color Doppler, high left-sided parasternal	0	<1.5	1.5–3	>3
Ductal velocity Vmax/Vmin ratio, m/s	PWD at pulmonary end of duct view	0	<1.5	1.5–2	>2
Antegrade LPA diastolic flow, cm/s	PWD within left pulmonary artery	0	<30	30–50	>50
Descending aorta diastolic velocity	PWD within descending aorta. High parasternal	Forward	Absent	Reverse	

* *adapted from Sehgal et al. (19): Maximum score = 11. Minimum score = 0*.

*LPA, left pulmonary artery; PWD, pulse wave Doppler*.

Median screening PDA score was 7 (IQR: 5–8.5) and 1 (IQR: 0–4) for the IB-T group and the CM group, respectively (*p* < 0.001). Median PDA score at the time ibuprofen treatment was administered was 9 (IQR: 8–9) (Tables 3, 4).

**Table 3:** Clinical data of the study population.

	**Conservative management (*n* = 42)**	**Ibuprofen treatment (*n* = 21)**	***p*-value**
Gestational age (weeks), median (IQR)	27.8 (26.7–28.4)	26 (24.6–26.6)	<0.001
Birth weight (g), median (IQR)	1056 (858–1056)	850 (660–957)	0.003
Maternal age (years), median (IQR)	33 (30–35.7)	31 (26.5–34)	0.2
5-min Apgar score <5, (IQR)	8 (6–8)	6 (4.5–8)	0.04
Cord pH, median (IQR)	7.30 (7.25–7.36)	7.34 (7.31–7.34)	0.02
Premature rupture of membranes >24 h, *n* (%)	13 (31)	2 (9)	0.07
Advanced resuscitation, *n* (%)^*^	15 (36)	14 (67)	0.03
Chorioamnionitis, *n* (%)^†^	18 (62)	14 (70)	0.7
Antenatal steroids, *n* (%)^z^	29 (70)	14 (67)	0.7
Multiple, *n* (%)	18 (43)	6 (29)	0.4
Male sex, *n* (%)	19 (45)	13 (62)	0.3
Surfactant therapy, *n* (%)	25 (60)	16 (76)	0.3
Postnatal age at screening NPE, median (IQR)	47.5 (24–48)	45 (24–56)	0.7
Platelet count (×10^3^/ml) at screening NPE, median (IQR)	215 (134.5–282.5)	241 (161–305)	0.2
Hemoglobin (g/dL) at screening NPE, median (IQR)	14.4 (12.6–15.7)	13.9 (12.8–15.3)	0.6
Postnatal age at PDA closure (days), median (IQR)	5 (2–15)	19 (9–34)	<0.001

* *intubation in the delivery room with or without assisted circulation*.

† *histological placental studies (n = 29, conservative management; n = 20, ibuprofen treatment)*.

z *full course*.

**Table 4:** Neonatologist Performed Echocardiography (NPE) variables.

	**Screening NPE**			**NPE at treatment**	
	**Conservative management (*n* = 42)**	**Ibuprofen treatment (*n* = 21)**	***p*-value**		*p*-value^*^
Transductal diameter, mm (median, IQR)	0 (0–1.5)	2 (1.7–2.6)	<0.001	2 (2.3–2.9)	0.01
Ductal velocity Vmax/Vmin ratio, m/s (median, IQR)	0 (0–1.4)	1.5 (1.2–2.2)	<0.001	2 (1.6–2.3)	0.02
Antegrade LPA diastolic flow, cm/s (median, IQR)	0.1 (0.08–0.18)	0.32 (0.17–0.38)	<0.001	0.36 (0.3–0.45)	0.006
Left atrial: aortic ratio (median, IQR)	1.6 (1.2–1.8)	1.7 (1.4–2)	0.3	1.9 (1.6–2.1)	0.01
LVO/SVC flow ratio (median, IQR)	2.2 (1.5–2.6)	2.4 (1.8–3.5)	0.07	3.7 (2.9–4.8)	0.02
E wave/A wave ratio (median, IQR)	0.75 (0.7–1)	0.8 (0.7–0.84)	0.8	0.8 (0.7–1)	0.08
Reverse descending aorta diastolic velocity, *n* (%)	2 (5)	11 (52)	<0.001	19 (90)	0.01
PDA Score (median, IQR)	1 (0–4)	7 (5–8.5)	<0.001	9 (8–9)	0.002

* *screening vs. treatment*.

The backward stepwise regression analysis showed that the best predictive model to estimate the likelihood of early spontaneous PDA closure included the gestational age [OR = 3.44 (95% CI 1.33–8.87)] and the screening PDA score [OR = 0.42 (95% CI 0.25–0.71)], according to the following equation,

Log (p/1-p) = −28.41 + 1.23^*^ gestational age −0.87^*^ PDA score at screening NPE.

Where, p is the probability of early spontaneous closure. Thus, for each unit increase in the PDA score at screening or in gestational age (in weeks), the likelihood of early spontaneous PDA closure decreases 58% or increases by 3.4 times, respectively.

## Discussion

The results of this analysis support screening PDA score at the second day of life in very preterm infants as a valuable tool to discriminate between two different groups of patients: those with a PDA pattern with high probability of early spontaneous closure during the first 2 weeks of life, and those who will develop unequivocal signs of hemodynamic significance related to a patent duct. In the backward stepwise regression analysis, only the gestational age improved the predictive capacity of the model. This is particularly relevant at extreme gestational ages as, according to our data, the likelihood of early spontaneous PDA closure at 24 weeks of gestation and PDA screening score of 5 would be around 4%, while at 29 weeks and the same score, it would reach 94%. Thus, the strength of PDA score is especially remarkable for intermediate gestational age groups, such as 26 weeks of gestation, where a score of 3 or 6 modifies dramatically the likelihood for early spontaneous closure, ranging from 72% to 16%, respectively. These findings highlight the importance of early personalized management of PDA instead of considering PDA as an all or none phenomenon. This personalized approach is essential to avoid overtreatment in infants with high chances of early spontaneous closure, and will focus on babies with high NPE screening scores that would be beneficiaries of early pharmacological prescription for PDA closure.

Early identification of this specific group may offer two main advantages. First, it has been reported that the efficacy of ibuprofen is inversely related to postnatal age at the time treatment is started; thus, the earlier the treatment the higher the likelihood of closure (14). Second, this predictive model also recognizes a subgroup of infants potentially most vulnerable to develop PDA-related morbidities, as a consequence of being exposed to a large blood shunting from the systemic to the pulmonary circulation. This population should be the focus of preventive strategies to minimize the hidden impact of PDA blood flow shifting from specific territories, either from the perspective of clinical management but also for research purposes.

Although, prolonged ductal patency may be associated with a variety of adverse outcomes (1–6), the pathophysiological mechanistic approach involving PDA hemodynamic changes has not been established yet. In addition, although some trials on early pharmacological therapy have shown a reduction in the short-term morbidities, such as pulmonary hemorrhage or severe IVH (9, 13), data on the long-term outcomes did not show benefits in the treatment group (7). The lack of long-term benefits in intervention trials on PDA closure could be explained by misclassification of the eligible population, as infants with PDA were selected for intervention independently of their individual PDA patterns, either if closing patterns were present or not.

We propose the early PDA score defined in this study as a tool for better selection of candidates for intervention in future trials on PDA treatment strategies. This early and objective score identified the population of low birth weight infants who received treatment for PDA closure, corresponding to one third of those who were systematically observed. Recent literature is aligned with our data, as spontaneous permanent closure of PDA by the end of the first week of life is reported in one third of the extremely low birth weight infants (16), and two thirds of them close their PDA by 2 months of life (20). These data have divided the scientific community in two currents with respect to PDA (treat or not treat) (7, 9, 11).

It is of note that active treatment with ibuprofen started at 69 h of birth in our study, although IB-T group screening NPE score was 7 at 42 h. The 27 h delay in ibuprofen treatment could have had a negative effect on treatment response, as the metabolism of ibuprofen has been shown to mature early in life (17).

Previous research focus on the management of PDA according to its capacity to cause harm (18, 19, 21). NPE offers information regarding hemodynamic PDA-related changes and allows the characterization of shunt volume over the lungs as well as the blood steal at the post-ductal circulation (15). Based on this information, different scores at random postnatal ages have been proposed (18, 19, 21) that have shown a relationship between shunt severity (higher PDA score) and IVH, NEC, BPD, or death (19, 21). In this retrospective study, screening NPE conducted on day 2 of birth only included in the PDA score the variables which predicted spontaneous early closure of PDA. Other parameters traditionally used to guide PDA treatment, such as left atrial to aortic ratio, LVO/SVC flow or E wave/A wave ratio, were excluded. These NPE variables may help to PDA disease staging but do not seem to be as useful for early in life treatment decision-making.

As opposite to previous reports (21), we decided not to use tissue Doppler imaging-derived variables to be included in our model because it was intended to define a more generalisable tool, easy to use by a wider population of neonatologists. Our score includes variables that define PDA size and flow (transductal diameter and ductal velocity), lung over-circulation (antegrade LPA diastolic flow), and systemic shunt effect (descending aorta diastolic velocity) according to the latest recommendations of the European Special Interest Group “Neonatologist Performed Echocardiography” (15). Neither platelet count nor the hemoglobin level (22) were found to be associated with PDA closing pattern in this study, so those were not included in the predictive model.

The main limitation of this study is its retrospective design so these results should be validated prospectively in a larger population. A greater predisposition of the neonatologist to treat infants with a lower gestational age and those with echocardiography-derived parameters indicating larger shunts is a matter of fact. As there was not a previously defined cut-off value to guide clinicians on when to start PDA treatment, only NPE variables statistically associated with treatment were included in the score that was retrospectively applied.

The strength, however, is to provide a tool to help clinicians in PDA decision making, by early identification of hsPDA that do not show closing patters, rather than to explore the association between PDA and morbidities. Future research will clarify whether prevention of PDA-related morbidities is feasible by effective early identification and treatment of hsPDA without closing patterns.

In conclusion, a predictive model of early spontaneous PDA closure that includes gestational age and the screening PDA score is proposed. This model may help clinicians in the decision-making process of PDA treatment but these results should be validated prospectively in a larger population. In addition, this model could be used to classify study groups for future intervention trials on ductal treatment that aim to prevent PDA related morbidities.

## Data Availability Statement

The raw data supporting the conclusions of this article will be made available by the authors, without undue reservation.

## Ethics Statement

The studies involving human participants were reviewed and approved by Ethics Committee for Human Studies at La Paz University Hospital. Written informed consent to participate in this study was provided by the participants' legal guardian/next of kin.

## Author Contributions

MB conceptualized and designed the study, performed some of the echocardiographic scans, drafted the initial manuscript, collected the information from the medical charts, performed the initial analyses, and approved the final manuscript as submitted. RS performed some of the echocardiographic scans, collected the information from the medical charts, and approved the final manuscript as submitted. AB performed some of the echocardiographic scans, collected the information from the medical charts, and approved the final manuscript as submitted. IL performed the statistical analysis, reviewed the manuscript, and approved the final manuscript as submitted. AP conceptualized and designed the study, drafted the initial manuscript, and approved the final manuscript as submitted. All authors contributed to the article and approved the submitted version.

## Conflict of Interest

The authors declare that the research was conducted in the absence of any commercial or financial relationships that could be construed as a potential conflict of interest.

## Abbreviations

PDA: patent ductus arteriosus
IVH: intraventricular hemorrhage
NEC: necrotizing enterocolitis
BPD: bronchopulmonary dysplasia
NPE: neonatologist performed echocardiographic studies
hsPDA: hemodynamically significant PDA
PWD: pulsed-wave Doppler
LPA: left pulmonary artery
LVO: left ventricular output
VTI: velocity time integral
SVC: superior vena cava.
